# Epigenetic dysregulation of energy homeostasis drives aortic valve stenosis that is treatable with metformin

**DOI:** 10.1172/jci.insight.188562

**Published:** 2025-09-09

**Authors:** Timothy J. Cashman, Sherin Saheera, Ashley E. Blau, Edith Mensah Otabil, Nouran Y. Nagy, Thomas D. Samenuk, Timothy P. Fitzgibbons, David D. McManus, Chinmay M. Trivedi

**Affiliations:** 1 Division of Cardiovascular Medicine, Department of Medicine;; 2 Program in Digital Medicine, Department of Medicine; and; 3 Department of Molecular, Cell, and Cancer Biology, University of Massachusetts Chan Medical School, Worcester, Massachusetts, USA.

**Keywords:** Cardiology, Clinical Research, Cardiovascular disease, Epigenetics, Mitochondria

## Abstract

Aortic valve stenosis is a progressive and increasingly prevalent disease in older adults, with no approved pharmacologic therapies to prevent or slow its progression. Although genetic risk factors have been identified, the contribution of epigenetic regulation remains poorly understood. Here, we demonstrated that histone deacetylase 3 (HDAC3) maintains aortic valve structure by suppressing mitochondrial biogenesis and preserving extracellular matrix integrity in valvular interstitial fibroblasts. Human stenotic valves displayed elevated acetylation of histone H3 at lysine 27 (H3K27ac) and reduced HDAC3 activity in diseased regions. Mice lacking HDAC3 in aortic valves developed aortic valve stenosis, disrupted collagen organization, increased H3K27ac, and premature mortality. Mechanistically, HDAC3 loss led to activation of nuclear hormone receptor–regulated mitochondrial gene programs, increased oxidative phosphorylation, and reactive oxygen species–induced damage. Treatment with metformin, a mitochondrial complex I inhibitor, restored redox balance, preserved collagen structure, and improved valve function in *Hdac3*-deficient mice. Supporting these experimental findings, retrospective clinical analysis revealed a significantly lower prevalence and slower progression of aortic valve stenosis in patients treated with metformin. These results uncovered a potentially previously unrecognized role for HDAC3 in coordinating epigenetic and metabolic homeostasis in the aortic valve, suggesting that targeting mitochondrial dysfunction may offer a therapeutic strategy for noncalcific aortic valve disease.

## Abstract 2

Epigenetic changes in nuclear DNA activate mitochondria abnormally, causing aortic valve stenosis; metformin emerges as a promising treatment.

## Graphical abstract

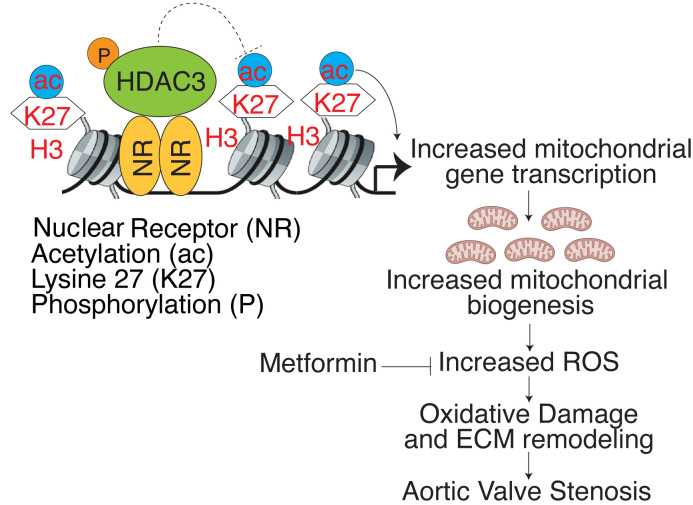

## Introduction

Aortic valve stenosis is the most prevalent form of valvular heart disease in aging populations, affecting up to 12% of individuals (1). It typically begins without symptoms and gradually progresses to severe obstruction of left ventricular outflow, ultimately leading to heart failure and death if left untreated (2). Currently, aortic valve replacement is the only effective therapy once symptoms appear, as no medical treatments exist to slow or prevent disease progression (3). Although several genetic risk factors have been identified, the molecular mechanisms — particularly those involving epigenetic regulation — remain poorly understood, limiting the development of targeted therapies. While fibrosis and extracellular matrix (ECM) remodeling are recognized features of aortic valve stenosis, the upstream drivers of these structural changes remain largely undefined.

Epigenetic mechanisms, including posttranslational histone modifications, are critical in regulating gene expression and maintaining cellular identity without altering the underlying DNA sequence (4). Among these, acetylation of histone H3 at lysine 27 (H3K27ac) is a well-established marker of active enhancers and promoters and serves as a key epigenetic signal controlling transcriptional activation (5). The opposing activities of acetyltransferases, EP300 and CBP, and histone deacetylase, HDAC3, dynamically balance this modification. HDAC3, a class I histone deacetylase, removes acetyl groups from H3K27, thereby repressing enhancer activity and downstream gene expression (6, 7). Phosphorylation further regulates HDAC3 function, enhancing its chromatin binding and deacetylase activity (8, 9). H3K27ac is a critical epigenetic marker of active enhancers and promoters, and its dysregulation can drive disease-specific gene expression (10). Although HDAC3 is a key deacetylase that targets H3K27ac, its role in regulating gene expression and tissue remodeling in aortic valve stenosis has not been investigated. Given the importance of epigenetic control in cardiovascular biology and the dynamic nature of enhancer and promoter regulation in tissue remodeling, elucidating the role of H3K27ac and HDAC3 in the aortic valve represents a critical step toward understanding the molecular basis of valvular disease.

Emerging evidence highlights a bidirectional relationship between mitochondrial metabolism and chromatin regulation, particularly through acetyl-CoA, which serves as a metabolic intermediate and a substrate for histone acetylation (11, 12). Although the influence of mitochondria on chromatin state is increasingly recognized, the reverse — how chromatin-modifying enzymes regulate mitochondrial function — remains poorly defined. HDAC3, though lacking a DNA-binding domain, is recruited to chromatin via interactions with transcription factors and co-repressor complexes (13). It functions as the enzymatic core of the nuclear receptor co-repressor complexes NCOR1 and NCOR2 (SMRT), which regulate nuclear hormone receptor activity and are essential for HDAC3’s histone deacetylase function, including deacetylation of H3K27 (14–16). Nuclear hormone receptors are central transcriptional regulators of mitochondrial biogenesis and oxidative metabolism, coordinating energy production in response to physiologic and pathologic stimuli (7, 17, 18). Co-repressor complexes tightly control their activity, yet how this regulation operates in valve interstitial cells remains unknown. Although increased oxidative stress and diminished antioxidant defenses have been documented in human stenotic valves (19, 20), the upstream epigenetic and transcriptional mechanisms that trigger these changes have not been defined, representing a critical gap in our understanding of aortic valve disease pathogenesis.

This study shows that HDAC3 preserves aortic valve structure and function by repressing mitochondrial biogenesis and maintaining ECM integrity in valvular interstitial fibroblasts. We demonstrate that tissue-specific loss of HDAC3 in murine aortic valves leads to increased H3K27ac at mitochondrial gene promoters, enrichment of nuclear hormone receptor motifs, excess mitochondrial activity, and ROS-mediated ECM remodeling — hallmarks of early valve pathology. These findings reveal a critical role for phosphorylated HDAC3 (p-HDAC3) in epigenetically constraining mitochondrial gene networks to preserve redox and structural homeostasis in the aortic valve. We further show that metformin, a clinically approved mitochondrial complex I inhibitor, restores metabolic balance and slows disease progression, highlighting a potential therapeutic strategy targeting this epigenetic-metabolic axis in noncalcific aortic valve disease.

## Results

### Conserved clinical traits in patients undergoing aortic valve replacement for severe stenosis.

We analyzed 8 human tricuspid aortic valves surgically excised from patients with severe, symptomatic disease to investigate the pathophysiology and underlying mechanisms of aortic stenosis. All patients met hemodynamic criteria for severe aortic stenosis, defined by an aortic valve area < 1.0 cm² and a mean transvalvular gradient ≥ 40 mmHg, as confirmed by echocardiography (Supplemental Figure 1; supplemental material available online with this article; https://doi.org/10.1172/jci.insight.188562DS1). The cohort included both men (62.5%) and women (37.5%), with ages ranging from 54 to 78 years (median 71.5), and most patients were classified as overweight based on BMI (Tables 1 and 2). Despite advanced valvular disease, none of the patients exhibited systolic heart failure, with preserved left ventricular ejection fractions averaging 60%–65%. Common comorbidities included hyperlipidemia and hypertension (87.5% each), often managed with ACEi, ARBs, or beta blockers. Coronary artery disease was present in 50% of patients, the majority were nonsmokers (87.5%), and 62.5% were on low-dose aspirin therapy. This clinically well-characterized patient cohort provided the basis for downstream tissue analysis and molecular profiling to explore disease mechanisms in human aortic valve stenosis.

### Focal epigenetic dysregulation of the HDAC3/H3K27ac axis in diseased human aortic valves.

**Figure 1:**
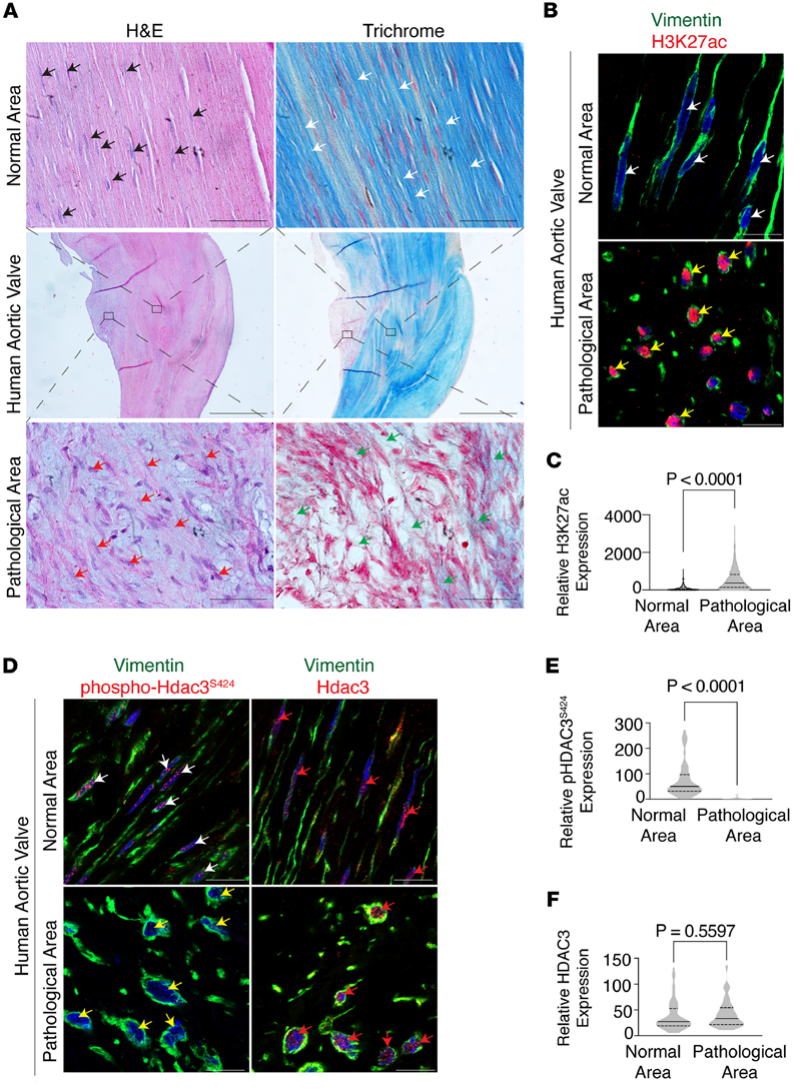
Histological and epigenetic alterations in human aortic valve stenosis. (**A**) Hematoxylin and eosin–stained (H&E; left) and trichrome-stained (right) sections of human aortic valves (*n* = 8) show organized collagen bundles with interspersed, parallel-aligned fibroblasts in normal regions (black and white arrows), in contrast with disrupted cellular alignment (red arrows) and reduced collagen content (green arrows) observed in pathological areas. Scale bar 50 μm (top and bottom row), 1,000 μm (middle row). (**B** and **C**) Coimmunofluorescence staining (**B**) and quantification (**C**) show increased H3K27ac expression (red, yellow arrows) in Vimentin^+^ valvular interstitial fibroblasts (green) in diseased regions compared with normal areas (white arrows). Hoechst nuclear counterstain (blue) of Vimentin^+^ valvular interstitial fibroblasts (green) shows spindle-shaped nuclei (white arrows) in normal areas compared with round or oval nuclei (yellow arrows) in pathological regions (yellow arrows). Unpaired *t* test with Welch’s correction. Scale bar 10 μm. (**D**–**F**) Coimmunofluorescence staining with Hoechst nuclear counterstain (**D**, blue) and quantification (**E** and **F**) show decreased phosphorylation of Hdac3 at Ser^424^ (green, **E**) in Vimentin^+^ valvular interstitial cells (yellow arrows) in pathological regions compared with normal areas (white arrows). Total Hdac3 (red) expression is similar between normal and pathological areas (red arrows, **F**). Unpaired *t* test with Welch’s correction. Scale bar 10 μm. Data represent median with interquartile range.

The study revealed that human aortic valves affected by stenosis exhibited distinct focal structural abnormalities. Pathological regions exhibited disrupted parallel alignment of interstitial Vimentin-positive fibroblasts, reduced collagen content, and fibroblasts with round or oval nuclei, in contrast with adjacent structurally preserved areas where fibroblasts displayed organized alignment and spindle-shaped nuclei (Figure 1, A and B). To assess epigenetic alterations, we performed immunostaining to evaluate histone H3 modifications at lysine residues 9 and 27, including acetylation, crotonylation, and methylation — key marks known to regulate gene transcription. Notably, interstitial fibroblasts within diseased regions showed an increase in H3K27ac compared with adjacent unaffected areas, while other histone modifications remained unchanged (Figure 1, B and C; data not shown). These findings suggest elevated H3K27ac is spatially associated with fibrotic remodeling in human aortic stenosis. Given that HDAC3, a class I HDAC, is a principal regulator of H3K27ac, we next examined its activity. Phosphorylation at serine 424 (Ser^424^) is required for HDAC3 deacetylase function (9). Immunostaining showed that while total HDAC3 expression remained unchanged, phosphorylation at Ser^424^ was lost in fibroblasts within diseased regions, indicating a localized impairment of HDAC3 enzymatic activity (Figure 1, D–F). These results collectively implicate epigenetic dysregulation, specifically via altered HDAC3-mediated H3K27 deacetylation, in the pathogenic regions of human aortic valve stenosis.

### Hdac3 prevents aortic valve stenosis by maintaining epigenetic and structural homeostasis in valve fibroblasts.

**Figure 2:**
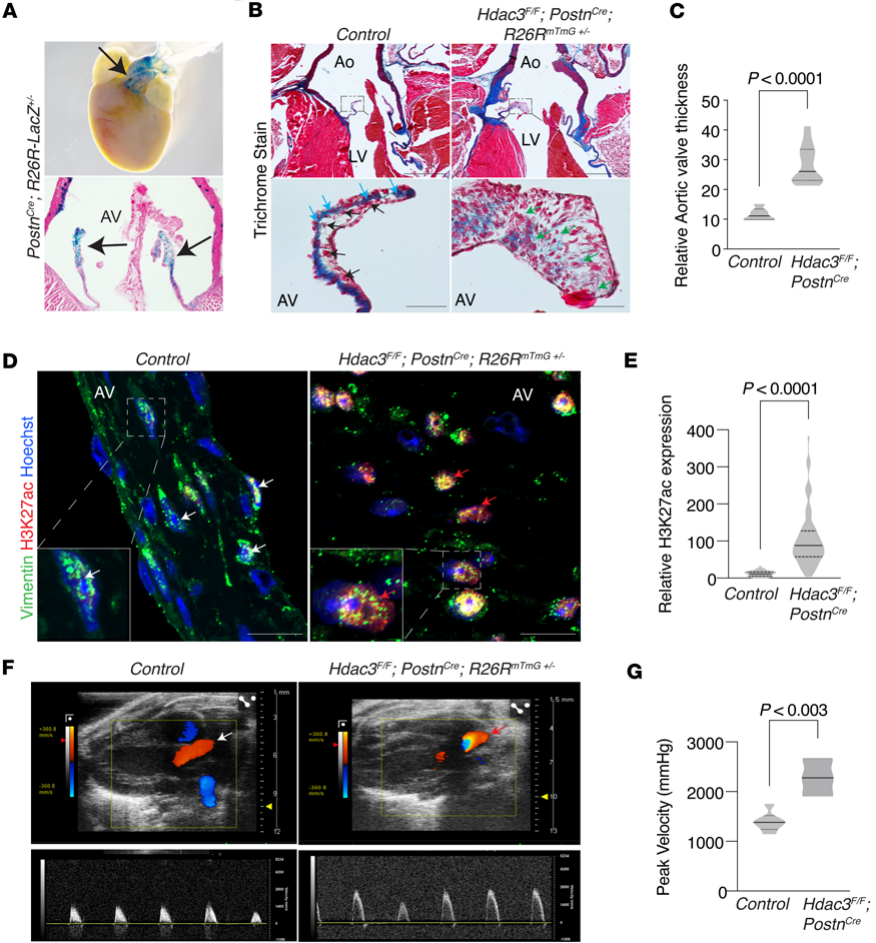
Genetic loss of Hdac3 in aortic valvular interstitial fibroblasts leads to aortic valve thickening, collagen disorganization, increased H3K27ac, and impaired valve function in mice. (**A**) LacZ-stained heart and frontal sections show *Postn*-Cre expression within outflow tract and aortic valves (black arrows). (**B** and **C**) Trichrome-stained sections of murine aortic valves (**B**, *n* = 6) and quantification (**C**) lacking Hdac3 display disorganized and reduced collagen deposition (**B**, green arrows), and valve thickening (**C**), compared with controls (black and blue arrows). Unpaired *t* test with Welch’s correction. Scale bar 500 μm (top row), 50 μm (bottom row). (**D** and **E**) Coimmunofluorescence staining with Hoechst nuclear counterstain (**D**, blue) and quantification (**E**) reveal increased H3K27ac expression (red) within Vimentin^+^ valvular interstitial cells (green, red arrows) of Hdac3-deficient aortic valves, compared with controls (white arrows). Hoechst nuclear counterstain (blue) of Vimentin^+^ valvular interstitial fibroblasts (green) shows spindle-shaped nuclei (white arrows) in control murine aortic valves compared with round or oval nuclei (red arrows) in Hdac3-deficient aortic valves (red arrows). Unpaired *t* test with Welch’s correction. Scale bar 10 μm. (**F** and **G**) Color doppler echocardiography (**F**) showing an increase in the peak velocity across the aortic valve in *Hdac3^fl/fl^* *Postn-Cre* mice (red arrow) compared with controls (white arrow). Echocardiographic assessment (**G**) of aortic valve function demonstrates elevated peak velocity across the aortic valve in *Hdac3^fl/fl^* *Postn-Cre* mice compared with controls. Unpaired *t* test. Data represent median with interquartile range. Ao, aorta; AV, aortic valve; LV, left ventrical.

Given that HDAC3 plays a unique role among class I HDACs in regulating H3K27ac, a histone mark critical for enhancer activity and gene transcription, it is important to develop a mouse model to mechanistically investigate its function in aortic valve stenosis and associated epigenetic remodeling. To this end, we generated a fibroblast-specific Hdac3-knockout model (*Hdac3^fl/fl^ Postn-Cre ROSA^mTmG+/–^*) to selectively ablate Hdac3 in valvular fibroblasts (Figure 2A). Mice with targeted Hdac3 deletion exhibited normal body size compared to controls (Supplemental Figure 2, A and B) but showed spontaneous lethality between 60 and 80 weeks of age (Supplemental Figure 2C). Histological analysis revealed a reduction in collagen deposition and increased valve thickness in Hdac3-knockout aortic valves (Figure 2, B and C). Interstitial fibroblasts within these valves displayed elevated H3K27ac, recapitulating the epigenetic profile observed in human stenotic aortic valves (Figure 2, D and E). Functional assessment via echocardiography demonstrated increased peak velocity across the aortic valve by 40 weeks of age in Hdac3-knockout mice, consistent with the development of aortic valve stenosis (Figure 2, F and G), while left ventricular ejection fraction remained comparable to controls (Supplemental Figure 2D). These findings establish Hdac3 as a critical regulator of valvular fibroblast epigenetic homeostasis and identify its loss as a driver of fibrotic remodeling and aortic valve stenosis.

### Hdac3 suppresses aberrant mitochondrial biogenesis in valve fibroblasts to maintain aortic valve function.

**Figure 3:**
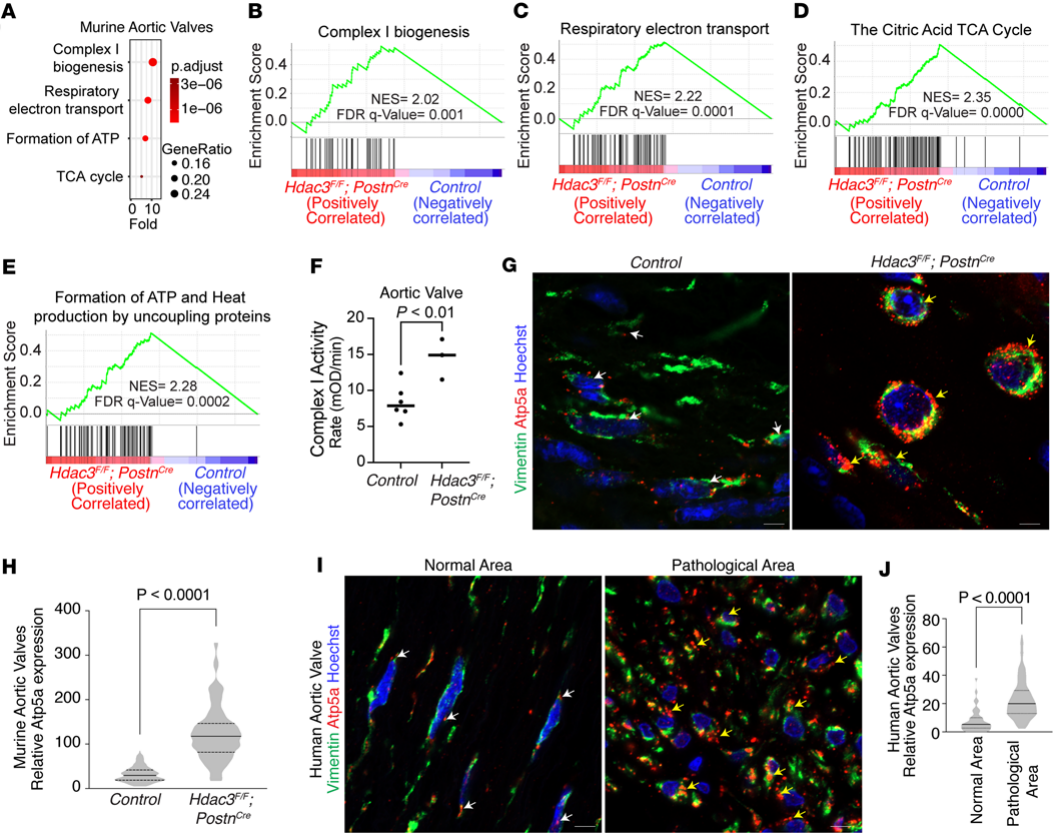
Loss of Hdac3 enhances mitochondrial biogenesis and activity in murine and human aortic valves. (**A**) Reactome pathway analysis reveals enrichment in the energy generation and mitochondrial biogenesis categories (*n* = 3). (**B**–**E**) GSEA of differentially regulated transcripts in murine aortic valves lacking Hdac3 compared with control (*n* = 3). *P* < 0.05. (**F**) Hdac3-knockout aortic valves exhibit an increase in mitochondrial complex I activity compared with control (*n* = 3). (**G**–**J**) Coimmunofluorescence staining with Hoechst nuclear counterstain (**G** and **I**; blue) and corresponding quantification (**H** and **J**) demonstrate increased mitochondrial density, indicated by elevated ATP5A expression (red; yellow arrows), in Vimentin^+^ valvular interstitial cells (green) in Hdac3-knockout murine aortic valves (**G**) and in pathological regions of human aortic valves (**I**), compared with control murine valves and normal human valve regions (**G** and **I**; white arrows). Unpaired *t* test with Welch’s correction. Data represent median with interquartile range. Scale bar 50 μm.

To investigate the molecular mechanisms underlying aortic valve stenosis following Hdac3 deletion, we performed bulk RNA-sequencing on aortic valves from control and Hdac3-knockout mice. Transcriptomic profiling revealed significant shifts in gene expression, with gene set enrichment analysis (GSEA) and Reactome pathway analysis identifying upregulation of pathways involved in mitochondrial energy production, including respiratory electron transport and oxidative phosphorylation (Figure 3, A–E, and Supplemental Figure 3, A–D). Conversely, pathways related to ECM organization were significantly downregulated, suggesting impaired structural integrity (Supplemental Figure 3A). Cytoscape-based network visualization further highlighted the interconnectivity among enriched mitochondrial pathways, including respiratory electron transport, energy metabolism, and mitochondrial biogenesis (Supplemental Figure 3, B and C). Functional validation using enzymatic assays verified increased mitochondrial activity in Hdac3-deficient valves compared with controls (Figure 3F). Consistent with these findings, immunofluorescence analysis revealed increased expression of ATP synthase F1 subunit alpha (ATP5A), a marker of elevated mitochondrial mass and metabolic activity, within Vimentin^+^ fibroblasts in both Hdac3-knockout murine valves and human stenotic aortic valves, suggesting enhanced mitochondrial biogenesis and a shift toward increased energy production in the stenotic valve microenvironment (Figure 3, G–J). These results suggest that loss of Hdac3 leads to metabolic reprogramming in valve fibroblasts, characterized by increased mitochondrial function and altered ECM gene expression, contributing to the pathogenesis of aortic valve stenosis.

### HDAC3 occupancy at mitochondrial biogenesis gene promoters restricts epigenetic activation in aortic valves.

**Figure 4:**
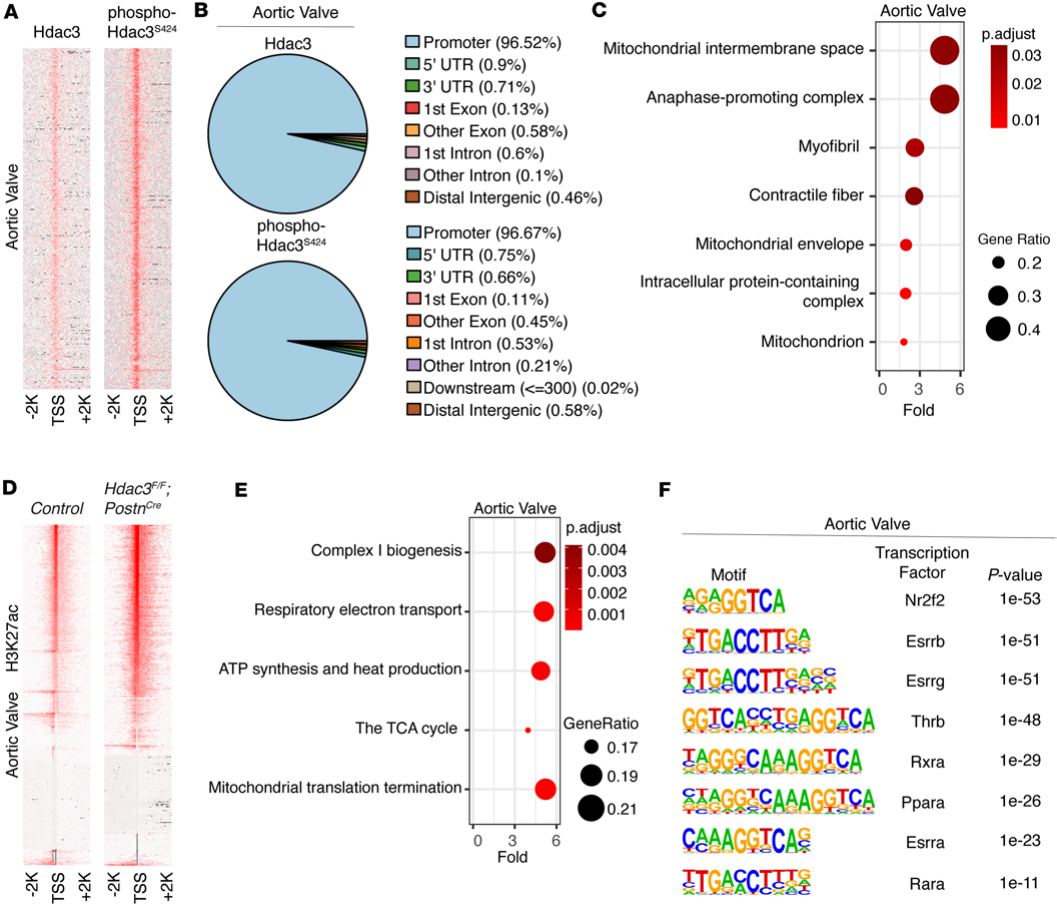
Hdac3 and phospho-Hdac3 localize to mitochondrial gene promoters to restrict H3K27ac enrichment in energy metabolism pathways. (**A**) Heatmaps display the enrichment of Hdac3 (left) and phospho-Hdac3^S424^ (right) at transcriptional start sites in murine aortic valves (*n* = 3). (**B**) Pie charts illustrate genome-wide occupancy of Hdac3 (top) and phospho-Hdac3^S424^ (bottom), showing enrichment at promoter regions in murine aortic valves. (**C**) Top enriched pathways from PANTHER cellular component analysis reveal Hdac3 and phospho-Hdac3^S424^ enrichment in mitochondrial categories within murine aortic valves. (**D**) Heatmaps show increased H3K27ac enrichment at transcriptional start sites in Hdac3-deficient murine aortic valves (right) compared with controls (left). (**E**) Top enriched pathways from Reactome pathway analysis of Hdac3-deficient murine aortic valves show increased H3K27ac enrichment in pathways related to energy production and mitochondrial biogenesis. (**F**) HOMER motif analysis identifies enrichment of nuclear receptor transcription factors at sites with increased H3K27ac enrichment in Hdac3-deficient murine aortic valves.

We performed Cleavage Under Targets and Tagmentation (CUT&Tag) to assess genome-wide occupancy of p-HDAC3 and total HDAC3 in adult murine aortic valves. Strikingly, the highest levels of HDAC3 and p-HDAC3 enrichment were observed at promoter regions of genes involved in mitochondrial biogenesis and oxidative phosphorylation, suggesting direct transcriptional repression of these energy-regulating pathways by HDAC3 (Figure 4, A–C, and Supplemental Figure 4). Notably, we did not observe significant enrichment of HDAC3 or p-HDAC3 at the promoters of genes involved in ECM organization despite their downregulation in Hdac3-deficient valves (data not shown). The lack of direct HDAC3 occupancy at these loci supports the interpretation that ECM gene expression changes are a secondary response to upstream metabolic or oxidative alterations. Reactome pathway analysis of H3K27ac CUT&Tag datasets from Hdac3-deficient aortic valves further supported this relationship, with the most significantly enriched pathways corresponding to mitochondrial biogenesis and oxidative phosphorylation (Figure 4, D and E, and Supplemental Figure 4). These findings indicate that HDAC3 deletion results in epigenetic activation of energy metabolism programs. To further elucidate the regulatory elements driving this activation, genome-wide motif analysis of H3K27ac-enriched promoter regions in Hdac3-knockout valves compared with controls revealed a strong enrichment of nuclear hormone receptor binding motifs, including Esrrg, Ppara, Thrb, and Esrra, which are known transcriptional activators of mitochondrial biogenesis (21–25) (Figure 4F). Together, these data demonstrate that HDAC3 and its phosphorylated form are preferentially enriched at mitochondrial gene promoters and that HDAC3 loss drives H3K27ac-dependent activation of mitochondrial biogenesis and oxidative metabolism pathways in the aortic valve.

### Hdac3 loss triggers ROS-dependent DNA damage and ECM-remodeling defects, which are mitigated by metformin.

**Figure 5:**
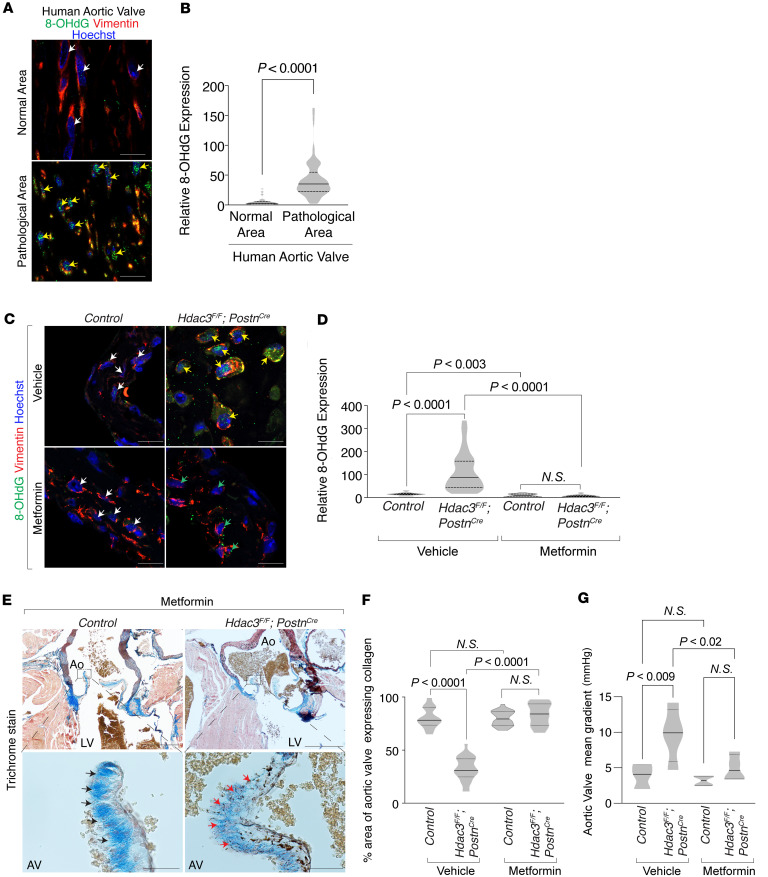
Metformin reduces oxidative DNA damage, preserves collagen structure, and improves aortic valve function in Hdac3-deficient stenotic valves. (**A** and **B**) Coimmunofluorescence staining (**A**) and quantification (**B**) reveal increased oxidative DNA damage, indicated by elevated 8-OHdG (green), in Vimentin^+^ valvular interstitial cells within pathological regions of human aortic valves (yellow arrows), compared with normal areas (white arrows). Unpaired *t* test with Welch’s correction. Scale bar 10 μm. (**C** and **D**) Coimmunofluorescence staining (**C**) and quantification (**D**) show increased 8-OHdG (green) in Vimentin^+^ valvular interstitial cells (red) of Hdac3-knockout aortic valves (yellow arrows) compared with controls (white arrows). Metformin treatment reduced 8-OHdG levels in Vimentin^+^ cells in both control (white arrows) and Hdac3-knockout valves (green arrows). Unpaired *t* test. Scale bar 10 μm. (**E** and **F**) Trichrome-stained sections of metformin-treated murine aortic valves and quantification (**F**) lacking Hdac3 display normal collagen deposition (red arrows) compared with controls (black arrows). Mann-Whitney test. Scale bar 500 μm (top row), 50 μm (bottom row). (**G**) The echocardiographic assessment shows improved mean gradient across the aortic valve in *Hdac3^fl/fl^* *Postn-Cre* mice treated with metformin, compared with untreated *Hdac3^fl/fl^* *Postn-Cre* mice. Ordinary 1-way ANOVA. Data represent median with interquartile range.

Building on our prior observation of aberrant mitochondrial biogenesis in Hdac3-knockout valvular interstitial fibroblasts, we hypothesized that elevated mitochondrial activity contributes to excessive ROS production, leading to oxidative DNA damage and subsequent fibrotic remodeling of the aortic valve. Coimmunofluorescence staining in human aortic valve specimens revealed elevated 8-hydroxy-2′-deoxyguanosine (8-OHdG) levels, a biomarker of oxidative stress, in Vimentin^+^ valvular interstitial fibroblasts within pathological regions compared with adjacent normal tissue, consistent with localized oxidative stress (Figure 5, A and B). Similarly, 8-OHdG expression was significantly increased in Vimentin^+^ valvular interstitial fibroblasts of Hdac3-knockout murine aortic valves, suggesting Hdac3 prevents mitochondrial ROS-mediated DNA damage (Figure 5, C and D). To test whether reducing mitochondrial ROS could mitigate this effect, we treated mice with metformin, a known inhibitor of mitochondrial complex I that suppresses ROS production (26, 27). Metformin treatment reduced 8-OHdG expression in valvular interstitial fibroblasts of both Hdac3-knockout and control valves (Figure 5, C and D). Furthermore, trichrome staining showed preservation of collagen architecture, and echocardiographic analysis demonstrated improved valve gradients in metformin-treated Hdac3-knockout mice (Figure 5, E–G, and Supplemental Figure 5). These findings support a model in which Hdac3 deficiency promotes mitochondrial oxidative stress and DNA damage in valvular interstitial fibroblasts, contributing to fibrotic valve remodeling, and identify metformin as a potential therapeutic agent to interrupt this pathogenic cascade.

### Reduced prevalence and attenuated progression of aortic valve stenosis in patients treated with metformin.

**Figure 6:**
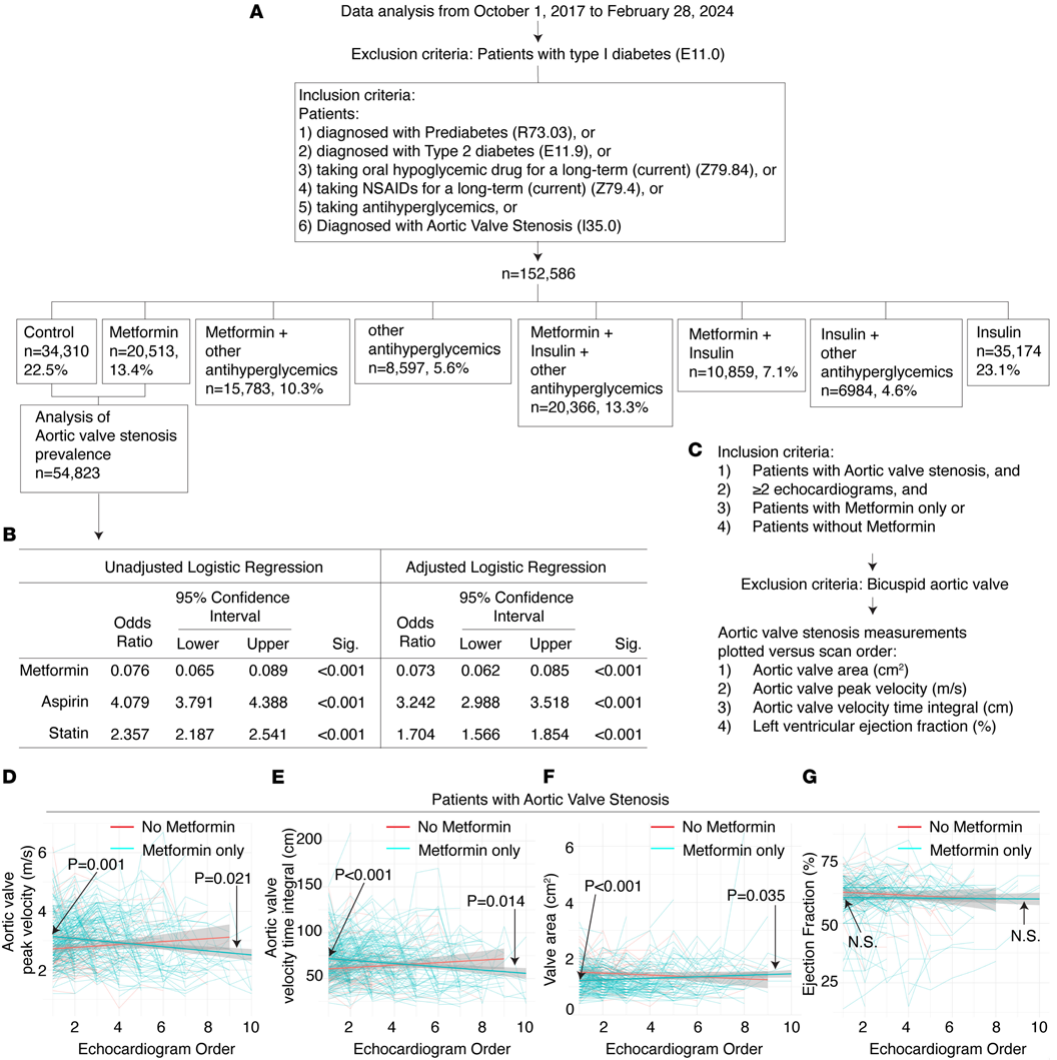
Metformin use is associated with reduced prevalence and slower progression of aortic valve stenosis in a retrospective clinical cohort. (**A**) Retrospective cohort analysis of 152,586 patients from October 1, 2017, to February 28, 2024, assessing the prevalence of aortic valve stenosis across medication groups. Comparative analysis of metformin-only users (*n* = 20,513) and nonmetformin controls (*n* = 34,310) was performed in a subset of 54,823 patients meeting inclusion criteria. (**B**) Patients taking metformin have lower odds of developing aortic valve stenosis compared with those not exposed. Categorical variables were analyzed using χ^2^ tests to examine associations, while continuous variables were assessed using *t* tests for mean comparisons between groups. (**C**–**G**) Longitudinal analysis of aortic valve function in patients with aortic valve stenosis and at least 2 echocardiograms, comparing those treated with metformin only versus those not on metformin (**C**). A linear mixed effects model was used to assess changes in peak velocity (**D**) (*n* = 1,063 from 262 patients), velocity time integral (**E**) (*n* = 1,054 from 260 patients), valve area (**F**) (*n* = 961 from 257 patients), and ejection fraction (**G**) (*n* = 810 from 250 patients) over time. The fixed effect results with interaction effect *P* values are shown. Patients with bicuspid aortic valves were excluded.

Metformin’s widespread clinical use and well-established safety profile, combined with our preclinical findings, provided a compelling rationale for conducting a retrospective clinical analysis to assess whether metformin use is associated with delayed onset or slowed progression of aortic valve stenosis in patients. A retrospective cohort analysis was conducted using patient data from October 1, 2017, to February 28, 2024, to assess whether metformin use is associated with a reduced prevalence of aortic valve stenosis (Figure 6A). The study included patients diagnosed with prediabetes or type 2 diabetes; those on long-term oral hypoglycemic agents, NSAIDs, or other antihyperglycemics; and those diagnosed with aortic valve stenosis. Patients with type 1 diabetes were excluded. Two groups were analyzed from a total cohort of 152,586 individuals: a control group of 34,310 patients not taking metformin or insulin and a metformin-only group of 20,513 patients. Compared with nonusers, metformin users were younger (mean age 59 vs. 65 years, *P* < 0.001) and had a higher average BMI (30.7 vs. 28.9, *P* < 0.001) (Table 3). Metformin users also had a higher mean HbA1c (6.3% vs. 5.8%, *P* < 0.001), reflecting more advanced glycemic dysregulation (Table 3). A greater proportion of metformin users had a diagnosis of type 2 diabetes (34.7% vs. 26.8%) (Table 3). These demographic and clinical differences were accounted for in adjusted analyses.

The prevalence of aortic valve stenosis was compared between these 2 groups to determine whether metformin use alone is associated with a lower rate of valve disease. Metformin users had a significantly lower prevalence of aortic valve stenosis (0.8% vs. 9.6%, *P* < 0.001) despite higher statin use and similar aspirin exposure (Table 3). Patients taking metformin exhibited significantly lower odds of developing aortic valve stenosis compared with those not taking it, with an odds ratio of 0.073 (95% CI: 0.062–0.085), after adjusting for potential risk factors, including aspirin and statin use (Figure 6B). In contrast, aspirin and statin intake were associated with significantly higher odds of aortic valve stenosis, with odds ratios of 3.242 (95% CI: 2.988–3.518) and 1.704 (95% CI: 1.566–1.854), respectively (Figure 6B).

Aortic valve stenosis is a progressive condition associated with significant morbidity and mortality, and there is a critical need to identify therapies that may slow its progression. To evaluate whether metformin use is associated with slower progression of aortic valve stenosis, we conducted a retrospective analysis of patients diagnosed with aortic valve stenosis who underwent at least 2 echocardiograms between October 1, 2017, and February 28, 2024 (Figure 6C). Patients were included if they were treated with metformin only or had no history of metformin use; those with bicuspid aortic valves were excluded. Serial echocardiographic parameters — including aortic valve area, peak velocity, velocity time integral, and left ventricular ejection fraction — were analyzed to assess disease progression over time (Figure 6C). A linear mixed model (LMM) revealed that patients treated with metformin had higher initial peak velocity and velocity time integral, but both declined significantly over time, whereas no significant changes were observed in the untreated group (Figure 6, D and E). Valve area, initially lower in the metformin group, increased significantly over time (Figure 6F). Ejection fraction was slightly lower at baseline in the metformin group, with no significant change during follow-up (Figure 6G). These results suggest that metformin use may be associated with a slower rate of aortic valve stenosis progression.

## Discussion

This study provides direct causal evidence linking aberrant mitochondrial biogenesis to the pathogenesis of aortic valve stenosis, offering a significant advancement in our understanding of the molecular drivers of this progressive disease. While previous observational studies have implicated mitochondrial dysfunction and oxidative stress in valve degeneration (19, 20, 28), the upstream regulatory mechanisms have remained largely undefined. Our findings identify HDAC3 as a key epigenetic suppressor of mitochondrial biogenesis in valvular interstitial fibroblasts. Loss of HDAC3 leads to excessive mitochondrial biogenesis, elevated production of ROS, and subsequent oxidative DNA damage — events that drive maladaptive ECM remodeling and contribute to valve narrowing. These results provide mechanistic context to earlier observations that stenotic human aortic valves exhibit elevated hydrogen peroxide and reduced superoxide dismutase levels in regions of pathology, reflecting redox imbalance driven by mitochondrial activity (29, 30). Additional support for our findings comes from animal and human studies, where increased ROS levels have been associated with accelerated valve calcification and stenosis (19, 20). Furthermore, genetic studies identifying mitochondrial haplotype H — characterized by higher mitochondrial activity — as a risk factor for aortic stenosis reinforce the link between mitochondrial output and disease severity (31, 32). Our study moves the field forward by demonstrating that mitochondrial dysregulation is not merely a consequence of valve disease but an upstream driver. By uncovering HDAC3 as a regulatory node in this pathway, our work opens avenues for targeted therapeutic strategies to modulate mitochondrial biogenesis and redox homeostasis to prevent or slow the progression of aortic valve stenosis.

Our study identifies metformin as a promising candidate for repurposing to reduce the prevalence and progression of aortic valve stenosis, revealing a potentially previously unrecognized mechanistic link between mitochondrial dysregulation and valvular remodeling. Although metformin is widely prescribed as a first-line therapy for type 2 diabetes and used globally by over 200 million individuals, its potential role in modifying structural heart disease has remained largely unexplored (33, 34). Through a combination of preclinical and retrospective clinical analyses, we demonstrate that metformin use is associated with a lower risk of developing aortic valve stenosis and a slower rate of disease progression. Mechanistically, we show that loss of HDAC3 leads to aberrant mitochondrial biogenesis, resulting in excess ROS, oxidative DNA damage, and impaired ECM maintenance — hallmarks of early fibrotic valve remodeling. Metformin mitigates these effects by inhibiting mitochondrial complex I, reducing ROS production, preserving ECM architecture, and improving valve function. Although ECM-associated genes were downregulated in Hdac3-deficient valves, we did not detect direct HDAC3 binding at their regulatory regions, suggesting these transcriptional changes are secondary to metabolic stress. Our study suggests that an aberrant oxidative environment suppresses gene expression programs essential for collagen organization, fibroblast contractility, and mechanosensitive signaling, thereby promoting valve stiffening and reduced compliance. By linking epigenetic control of mitochondrial homeostasis to ECM remodeling and demonstrating that this pathway is therapeutically targetable, our findings not only advance the mechanistic understanding of aortic valve stenosis but also support the rationale for clinical trials investigating metformin in early-stage, noncalcific valvular disease.

This study reveals a potentially new and critical role for p-HDAC3 in maintaining aortic valve homeostasis by epigenetically repressing mitochondrial biogenesis and preserving ECM integrity in valvular interstitial fibroblasts. Although our previous studies have established roles for HDAC3 in cardiac development and lymphatic vascular function, its specific contribution to aortic valve disease has not been investigated (15, 35). Here, we demonstrate that p-HDAC3 is highly enriched at the promoters of mitochondrial genes in the valve, where it restricts H3K27ac to suppress oxidative phosphorylation and energy metabolism pathways. Consistent with this mechanism, reduced phosphorylation of HDAC3 in human stenotic aortic valves corresponds with elevated H3K27ac levels in pathogenic regions, indicating a loss of deacetylase activity and epigenetic control. Importantly, our H3K27ac CUT&Tag and motif enrichment analyses identified significant upregulation of nuclear hormone receptor binding motifs — including Esrrg, Ppara, Thrb, and Esrra — in mitochondrial gene promoters and enhancers in Hdac3-deficient valves. These nuclear receptors are ligand-regulated transcription factors that dynamically regulate mitochondrial biogenesis and fuel metabolism in response to physiologic and pathologic signals (36–39). Prior studies have shown that overactivation of *Esrrg* or *Ppara* enhances mitochondrial oxidative capacity and increases susceptibility to oxidative stress, particularly when antioxidant systems are overwhelmed (22, 36, 39). Likewise, dysregulated *Thrb* and *Esrra* signaling has been linked to cardiac hypertrophy and energetic imbalance (21). Our findings suggest that HDAC3 functions as an epigenetic gatekeeper, limiting inappropriate activation of this nuclear receptor network to prevent mitochondrial overdrive and structural valve degeneration. This work significantly advances the field by establishing HDAC3 — and its phosphorylation state — as a key chromatin-bound regulator of mitochondrial transcription in the aortic valve, offering insights into how HDACs modulate metabolic-epigenetic crosstalk in cardiovascular disease.

## Methods

### Sex as a biological variable.

The study included both male and female mice and human patients. No sex-specific differences were observed in disease phenotype or outcomes.

### Human samples and clinical data analysis.

Tricuspid aortic valves were collected from patients undergoing surgical valve replacement at UMass Memorial Medical Center. Following receipt of patient informed consent, valves were bisected: one half was flash-frozen at –80°C, and the other was fixed in 4% paraformaldehyde for histologic analysis. Samples and data were deidentified prior to analysis. To investigate the clinical effects of hypoglycemic agents on aortic valve disease, a retrospective cohort was generated from electronic health records spanning October 1, 2017, to February 28, 2024. Inclusion criteria included diagnosis of prediabetes or type 2 diabetes mellitus, long-term use of NSAIDs or oral hypoglycemics, and/or diagnosis of valvular heart disease, including aortic valve stenosis. Patients with type 1 diabetes were excluded. For prevalence analysis, we compared patients with documented long-term metformin-only use and those with no metformin exposure. Clinical and demographic characteristics were similar between groups (Table 3). For progression analysis, patients diagnosed with aortic valve stenosis, and at least 2 echocardiograms were included. Key echocardiographic parameters — valve area, peak velocity, velocity time integral, and ejection fraction — were extracted from imaging reports. Longitudinal changes in these measurements were analyzed using an LMM, which accounts for repeated observations within individuals by incorporating random intercepts and slopes. Fixed effects included time (scan order), treatment group (metformin vs. no metformin), and their interaction to evaluate differences in progression between groups. This approach allowed for individualized change modeling while assessing group-level differences in disease trajectory.

### Mouse models.

Hdac3^fl/fl^ mice (40) were bred with Postn-Cre and mT/mG reporter mice (41) to generate fibroblast-specific Hdac3-knockout and control littermates. Genotyping was performed by PCR using validated primer sets: *Hdac3*: forward 5′-TGGTGGTGAATGGCTTTAATC-3′, reverse 5′-TAACGGGAGCAGAACTCGAA-3′ *Cre*: forward 5′-ATTCTCCCACCGTCAGTACG-3′, reverse 5′-CGTTTTCTGAGCATACCTGGA-3′ *mT/mG*: forward 5′-CTCTGCTGCCTCCTGGCTTCT-3′, mutant reverse 5′-TCAATGGGCGGGGGTCGTT-3′, WT reverse 5′-CGAGGCGGATCACAAGCAATA-3′.

### Histology and immunohistochemistry.

Mouse hearts and human valve tissues were fixed in 4% paraformaldehyde at 4°C overnight, processed through ethanol dehydration, embedded in paraffin, and sectioned at 8 μm. H&E staining was performed using Harris hematoxylin solution, modified (Sigma, catalog HHS32-1L) and Eosin Y (Fisher, catalog 22-050-198). Paraffin sections underwent antigen retrieval using the 2100 Antigen Retriever (Aptum Biologics) for immunohistochemistry, followed by blocking with 10% donkey serum. Sections were incubated with primary antibodies overnight at 4°C and then with secondary antibodies (1:500) and Hoechst (1:1,000) for nuclear staining before mounting in Vectashield. To detect oxidative DNA damage, sections were incubated with anti–8-OHdG followed by fluorescent secondary antibodies from ThermoFischer Scientific: Donkey anti-rabbit IgG (H+L) Alexa Fluor 568 (catalog A10042), Donkey anti-goat IgG (H+L) Alexa Fluor 568 (catalog A11057), Donkey anti-rabbit IgG (H+L) Alexa Fluor 488 (catalog A-21206), Donkey anti-rat IgG (H+L) Alexa Fluor 488 (catalog SA510026), Donkey anti-goat IgG (H+L) Alexa Fluor 488 (catalog SA510086), Donkey anti-mouse IgG (H+L) Alexa Fluor 488 (catalog A21202), Donkey anti-mouse IgG (H+L) Alexa Fluor 546 (catalog A10036), Donkey anti-rat IgG (H+L) Alexa Fluor 594 (catalog A21209). Colocalization with Vimentin was used to identify valvular fibroblasts. Quantification was performed using ImageJ (NIH) on multiple high-power fields per section. Masson’s trichrome staining was performed to assess collagen content. The collagen-positive area was quantified relative to the total valve area using ImageJ.

### Antibodies.

Primary antibodies included HDAC3 (Santa Cruz Biotechnology SC11417, Abcam ab7030), p-HDAC3 (Cell Signaling Technology, 3815s), H3K27ac (Abcam, ab4729), ATP5A (Abcam, ab14748), Vimentin (Thermo Fisher Scientific MA5-11883, Cell Signaling Technology 5741), and 8-OHdG (Santa Cruz Biotechnology, SC393871).

### CUT&Tag assay.

Aortic valves from 12-week-old control and Hdac3-knockout mice were used for CUT&Tag (42) following Active Motif’s protocol (catalog 53160) with minor modifications (43). Nuclei were isolated, bound to Concanavalin A beads, and incubated overnight with antibodies targeting p-HDAC3, HDAC3, H3K27ac, or IgG control (Cell Signaling Technologies, catalog 66362S). Indexed libraries were prepared and sequenced on an Illumina MiSeq platform with 25 bp paired-end reads. Reads were trimmed, aligned to the mm10 genome using Bowtie2 (44), and filtered for quality (45). Peaks were called with MACS, annotated with ChIPseeker (46), and visualized using HOMER (47) and DolphinNext (48). Motif enrichment analysis of H3K27ac peaks was performed using HOMER. Differential peaks between knockout and control samples were analyzed for transcription factor binding site enrichment, and significantly enriched motifs were mapped to known nuclear hormone receptors.

### RNA sequencing.

GFP-expressing aortic valve tissue was dissected from 3 knockout and 3 control mice, pooled by genotype, and processed for RNA extraction (RNeasy Mini Kit, QIAGEN). Libraries were prepared and sequenced (NovaSeq 6000 PE150, Novogene). Data were aligned to mm10 using STAR, quantified with RSEM, and analyzed for differential expression in DEBrowser (https://github.com/UMMS-Biocore/debrowser). Gene ontology and pathway analysis were performed using the PANTHER classification system.

### Imaging.

H&E-stained sections were imaged using a Nikon Eclipse 80i microscope with Plan Fluor objectives and NIS-Elements software. Immunofluorescence was performed using a ZEISS LSM800 Airyscan confocal microscope with differential interference contrast optics and 405/488/561/640 nm laser modules. High-resolution images were acquired with 20× and 63× Plan-Apochromat objectives.

### Mitochondrial complex I activity assay.

Complex I activity was measured in mouse aortic valve lysates using the Abcam Complex I Enzyme Activity Assay Kit (ab109721). Following homogenization and protein extraction, lysates were incubated on antibody-coated plates, and enzyme activity was determined by measuring absorbance at 450 nm.

### Metformin treatment in mice.

Metformin (1.25 mg/mL) was administered in drinking water for 6 weeks. Based on an average daily intake of 5 mL/mouse, the estimated dose was ~187.5 mg/kg/d, equivalent to ~1,200 mg/d in humans. Fresh metformin solution was replaced every 3–4 days.

### Echocardiography.

Echocardiographic evaluation was performed under isoflurane sedation using the VisualSonics Vevo 3100 system. Functional parameters included aortic valve peak velocity, mean gradient, valve area, ejection fraction, ventricular dimensions, and wall thickness.

### Statistics.

Data are presented as mean ± SD. Comparisons between the 2 groups were made using unpaired 2-tailed *t* tests. Categorical variables were assessed by χ^2^ test. Multivariable logistic regression was used to assess the association between metformin use and aortic valve stenosis, adjusting for potential confounders. Differences in 8-OHdG and collagen content were analyzed using 2-tailed *t* tests across biological replicates. Statistical significance was set at *P* < 0.05. Analyses were performed using GraphPad Prism 10 and IBM SPSS Statistics version 29.

### Study approval.

All animal protocols were approved by the Institutional Animal Care and Use Committee at the University of Massachusetts Chan Medical School. Human tissue collection and clinical data review were approved by the Institutional Review Board, with written informed consent obtained from all patients.

### Data availability.

Sequencing data are available in the National Center for Biotechnology Information Gene Expression Omnibus under accession numbers GSE302550 and GSE302552, in the Supporting Data Values Excel file, or upon request from the corresponding author.

## Author contributions

Conceptualization was contributed by CMT. Methodology was contributed by TJC, SS, AEB, EMO, TDS, NYN, TPF, DDM, and CMT. Investigation was contributed by TJC, SS, AEB, NYN, TPF, DDM, and CMT. Supervision was contributed by TPF, DDM, and CMT. Writing of the original draft was contributed by TJC and CMT. Review and editing were contributed by TJC, SS, TPF, DDM, and CMT.

## Supplementary Material

Supplemental data

Supporting data values

## Figures and Tables

**Table 1:**
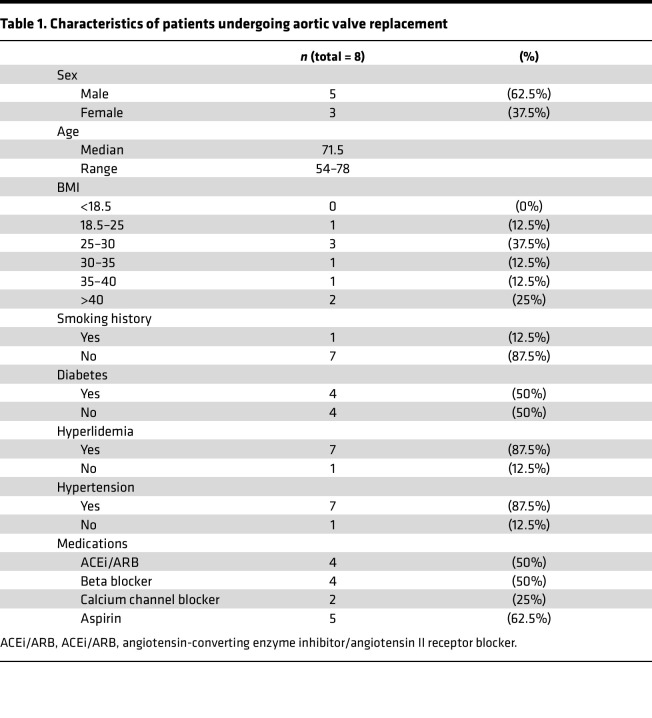
Characteristics of patients undergoing aortic valve replacement

**Table 2:**
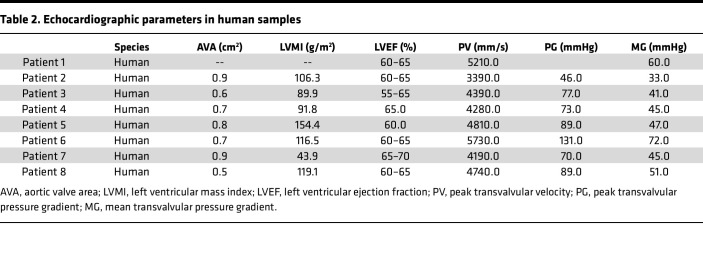
Echocardiographic parameters in human samples

**Table 3:**
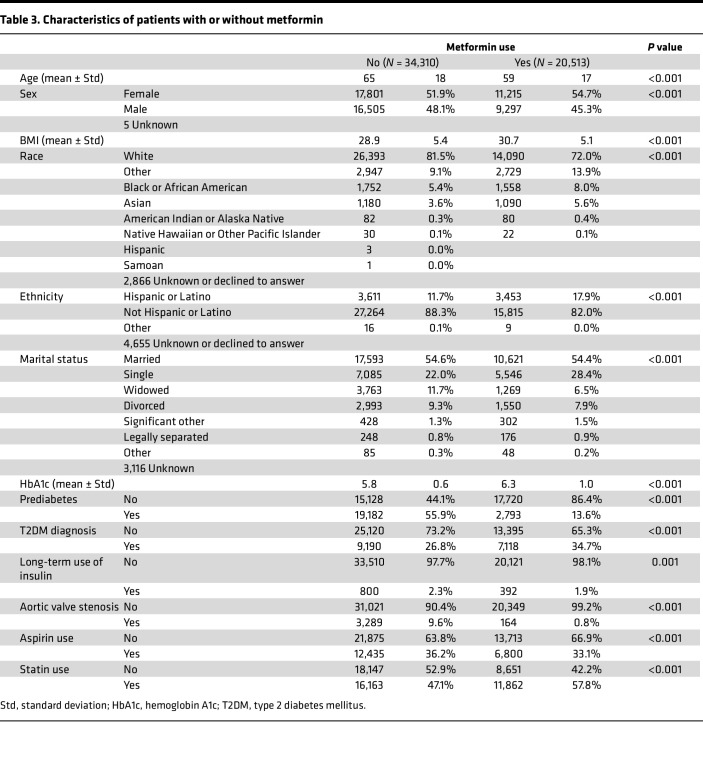
Characteristics of patients with or without metformin

## Version 1 09/09/2025

Electronic publication
