# Supplementary material for: Epigenetic dysregulation of energy homeostasis drives aortic valve stenosis that is treatable with metformin
Source: JCI Insight. 2025 Sep 9;10(17):e188562. doi: 10.1172/jci.insight.188562 (PMC12487670; doi:10.1172/jci.insight.188562)
Supplement: Supplemental data [file jciinsight-10-188562-s122.pdf]

Supplemental Data:

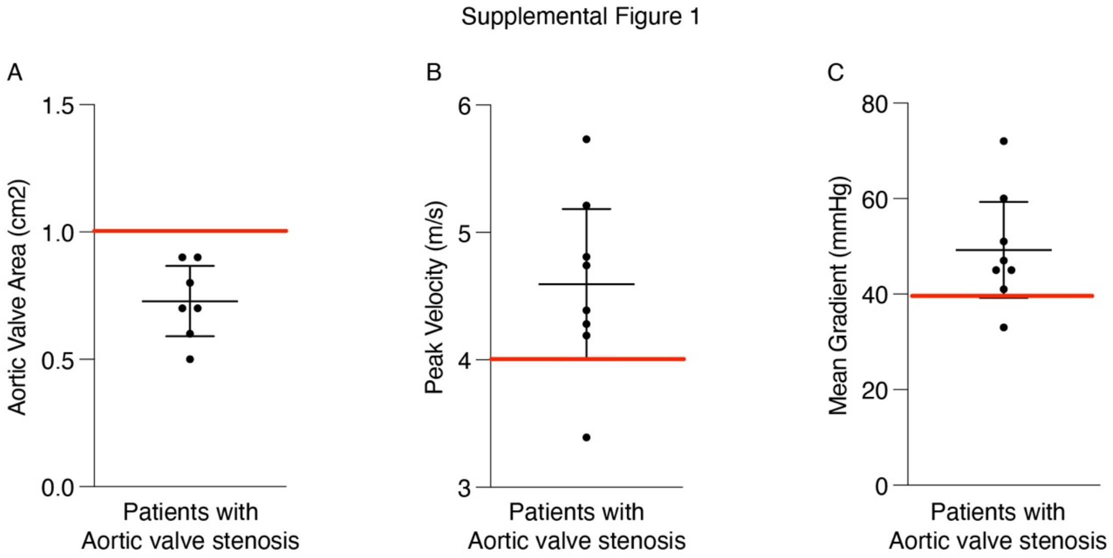

Supplemental figure 1: **(A-C)** Echocardiographic aortic valve area (A), peak velocity (B), and mean gradient (C) in human patients with aortic valve stenosis. Red line depicts severity cutoff. Aortic valve area less than 1 cm^2 is considered severe (A).

Supplemental Figure 2

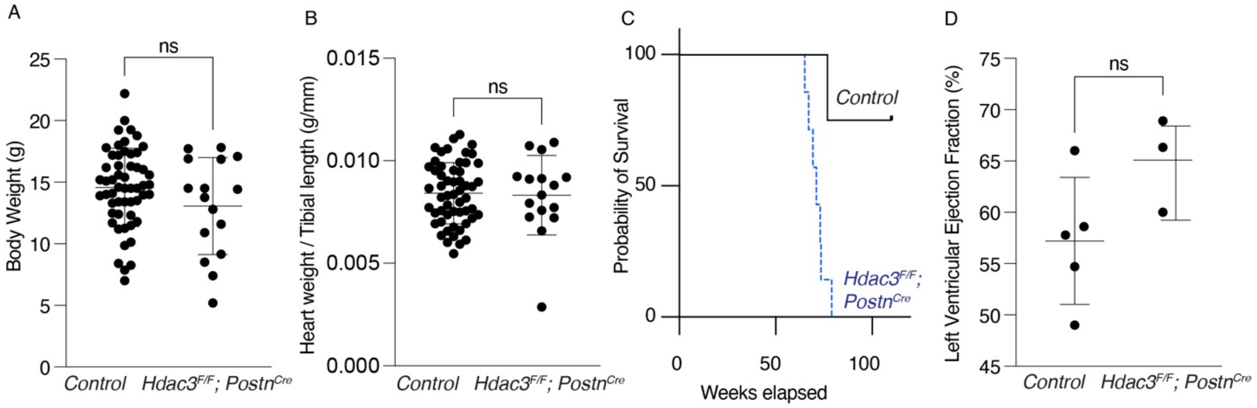

Supplemental Figure 2: (A-B) Body weight (A) and Heart weight / tibial length ratio (B) measurements of *Hdac3*$^{F/F}$; *Postn-Cre* mice compared to controls. Unpaired t test with Welch's correction. **(C)** Kaplan-Meier survival analysis shows complete lethality in *Hdac3*$^{F/F}$; *Postn-Cre* mice. Logrank (Mantel-Cox) test ($P < 0.009$) **(D)** Echocardiographic assessment of aortic valve function demonstrates normal left ventricular ejection fraction in *Hdac3*$^{F/F}$; *Postn-Cre* mice compared to controls. Unpaired t test. ns = not significant ($P > 0.05$).

Supplemental Figure 3

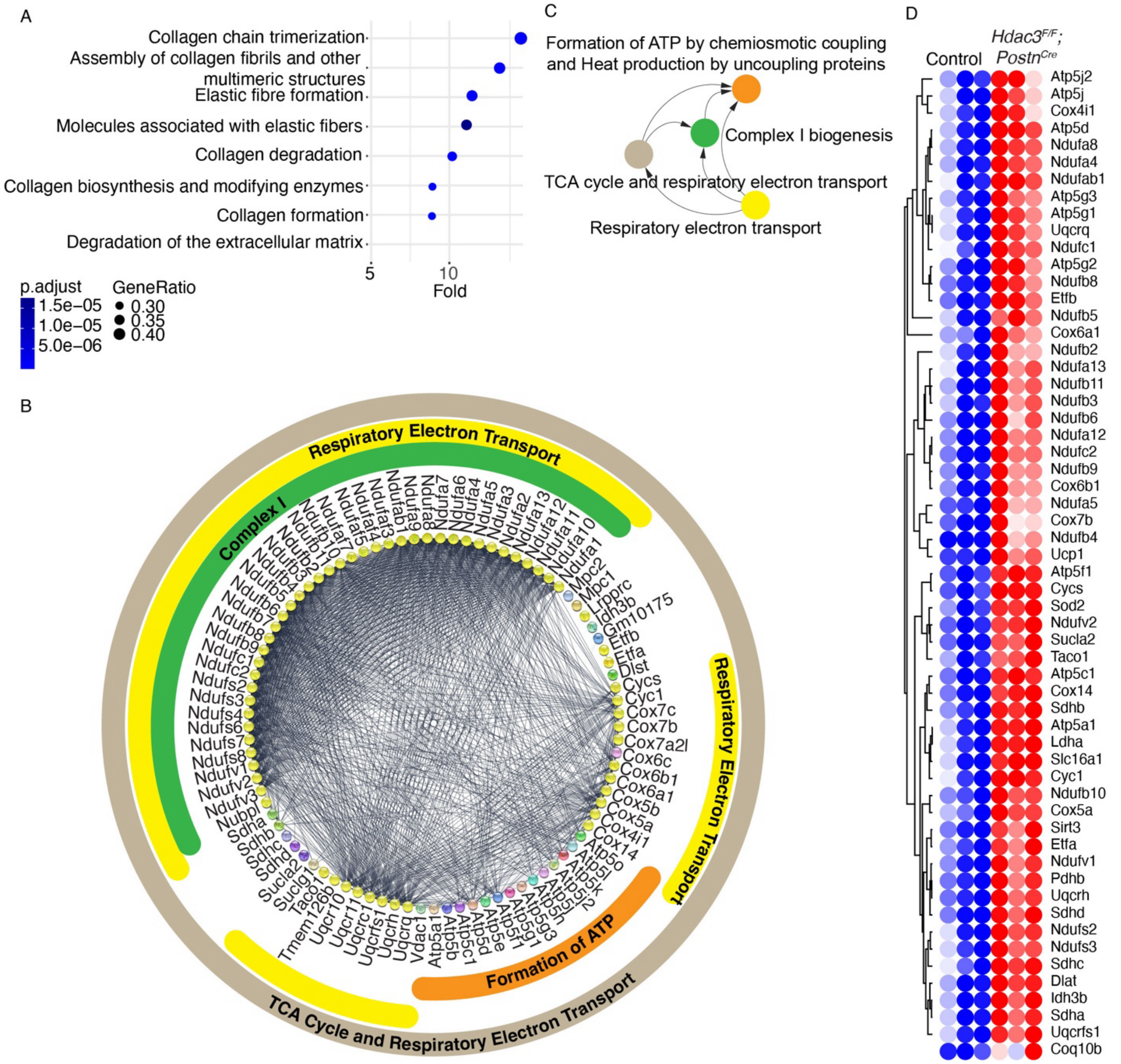

Supplemental Figure 3. (A) Reactome pathway analysis of downregulated genes reveals enrichment in the collagen formation and assembly categories (n = 3). **(B-C)** Cytoscape network analysis of the Reactome pathways reveals interconnected relationships between enriched categories, including respiratory electron transport, energy generation, and mitochondrial

biogenesis. **(D)** Heatmap of top differentially regulated transcripts within GO biological process categories of energy regulation (n = 3).

Supplemental Figure 4

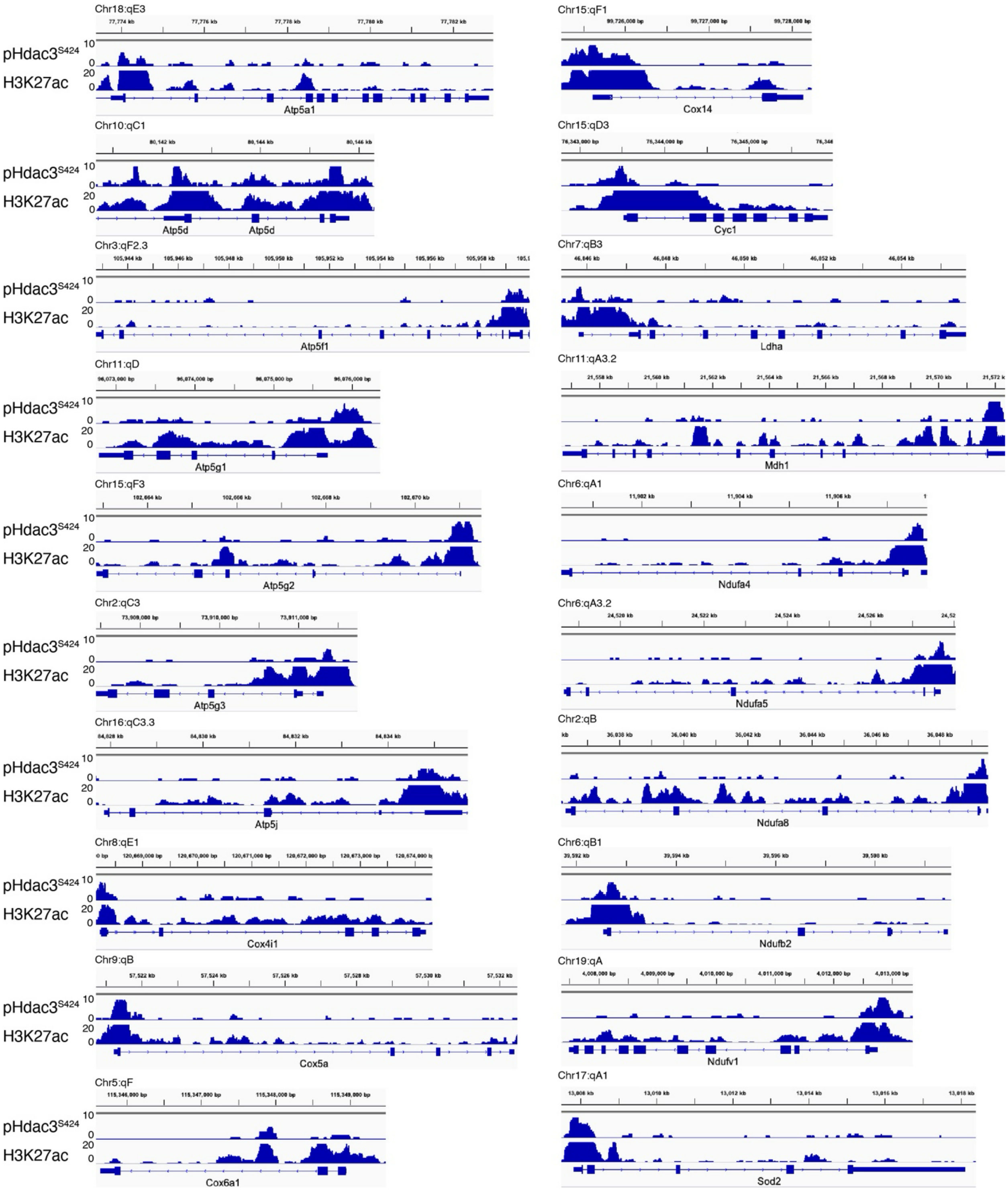

Supplemental figure 4. CUT&Tag analyses of pHdac3^{Ser424} or H3K27ac showing enrichment at the transcriptional start sites of top differentially regulated transcripts within GO biological process categories of energy regulation. The top track shows pHdac3^{Ser424}-occupied DNA sequences (blue peaks) in control murine aortic valves normalized to IgG ($P < 0.05$). The bottom track shows H3K27ac-occupied DNA sequences (blue peaks) in Hdac3-knockout aortic valves normalized to H3K27ac enrichment in control murine aortic valves ($P < 0.05$).

Supplemental Figure 5

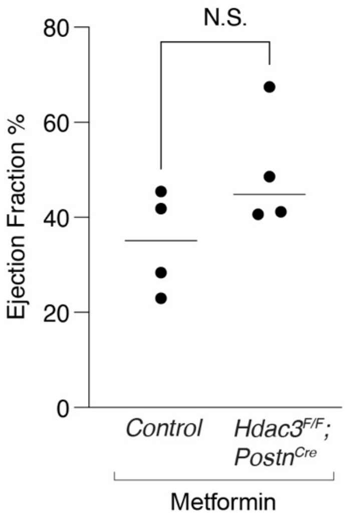

Supplemental figure 5. Echocardiographic assessment of aortic valve function demonstrates normal left ventricular ejection fraction in *Hdac3*$^{F/F}$; *Postn-Cre* mice treated with Metformin compared to controls. Unpaired t test. ns = not significant ($P > 0.05$).
